# Delimiting species, revealing cryptic diversity in Molytinae (Coleoptera: Curculionidae) weevil through DNA barcoding

**DOI:** 10.1093/jisesa/ieae083

**Published:** 2024-09-30

**Authors:** Jinliang Ren, Runzhi Zhang

**Affiliations:** Key Laboratory of Zoological Systematics and Evolution, Institute of Zoology, Chinese Academy of Sciences, Beijing 100101, China; College of Life Science, University of Chinese Academy of Sciences, Beijing 100049, China; Key Laboratory of Zoological Systematics and Evolution, Institute of Zoology, Chinese Academy of Sciences, Beijing 100101, China; College of Life Science, University of Chinese Academy of Sciences, Beijing 100049, China

**Keywords:** biodiversity, cryptic species, DNA barcode, Molytinae, species delimitation

## Abstract

The subfamily Molytinae (Coleoptera: Curculionidae), being the second largest group within the family Curculionidae, exhibits a diverse range of hosts and poses a serious threat to agricultural and forestry industries. We used 1,290 cytochrome c oxidase subunit I (COI) barcodes to assess the efficiency of COI barcodes in species differentiation and uncover cryptic species diversity within weevils of Molytinae. The average Kimura 2-parameter distances within species, genus, and subfamily were 2.90%, 11.0%, and 22.26%, respectively, indicating significant genetic differentiation at both levels. Moreover, there exists a considerable degree of overlap between intraspecific (0%–27.50%) and interspecific genetic distances (GDs; 0%–39.30%). The application of Automatic barcode gap discovery, Assemble Species by Automatic Partitioning, Barcode Index Number, Poisson Tree Processes (PTP), Bayesian Poisson Tree Processes (bPTP), and jMOTU resulted in the identification of 279, 275, 494, 322, 320, and 279 molecular operational taxonomic units, respectively. The integration of 6 methods successfully delimited species of Molytinae in 86.6% of all examined morphospecies, surpassing a threshold value of 3% GD (73.0%). A total of 28 morphospecies exhibiting significant intraspecific divergences were assigned to multiple MOTUs, respectively, suggesting the presence of cryptic diversity or population divergence. The identification of cryptic species within certain morphological species in this study necessitates further investigation through comprehensive taxonomic practices in the future.

## Introduction

The identification and delimitation of species through morphology-based traditional taxonomy is a crucial approach for exploring biodiversity (Li et al. 2023). However, the accurate and rapid identification of species based on conventional morphological methods sometimes pose challenges due to the morphological resemblance among closely related species (Meregalli et al. 2020), interference from different life stages (Hajibabaei et al. 2006), declining numbers of taxonomists (Miller 2007, Ma et al. 2022), the presence of species complexes and the occurrence of cryptic diversity (Hebert et al. 2004). Thus, the completion of biodiversity inventory using traditional morphological methods alone is nearly impossible due to the vast number of insect groups.

With advancements in molecular technology, Hebert et al. (2003) proposed that DNA barcoding could serve as a fundamental component of a global bioidentification system. The taxonomic names are assigned using this method, which relies on the standard genetic markers, such as the mitochondrial cytochrome oxidase subunit I (COI), by comparing their sequences with those in reference databases (Hebert and Gregory 2005). The efficacy of this approach has been successfully demonstrated across various taxonomic groups, including birds (Charles et al. 2004), fish (Ward et al. 2005, Sheraliev and Peng 2021), and, subsequently, insects (Park et al. 2011, Pramual and Adler 2014, Nzelu et al. 2015, Dinca et al. 2021, da Conceição Abreu Bandeira et al. 2023). In addition, COI barcodes have also been demonstrated to be suitable for unveiling biogeographic patterns (Greenstone et al. 2011), elucidating cryptic species (Hebert et al. 2004, Gopurenko et al. 2022, Hew et al. 2023), revealing biodiversity (Lahaye et al. 2008, Ge et al. 2021, Chimeno et al. 2022), exploring population genetics (Li et al. 2019, Fernandez et al. 2022), discovering new pests (Yang et al. 2021), and reconstructing phylogenetic relationships at the species level (Grebennikov 2014, 2022).

Weevils (Coleoptera: Curculionoidea) represent one of the most diverse beetle groups, encompassing over 62,000 identified species (5,800 genera) (Oberprieler et al. 2007). The subfamily Molytinae stands as the second largest subfamily within Curculionoidea (Oberprieler et al. 2007, Shin et al. 2018). The identification and classification efforts of Molytinae have faced significant challenges due to the vast number of species and certain poorly studied groups (Oberprieler et al. 2007, Grebennikov 2020, Kumar et al. 2023). Moreover, Molytinae exhibits a diverse range of hosts encompassing dicots, monocots (particularly palms), as well as gymnosperms (conifers and cycads) (Kuschel 1987, Alonso-Zarazaga and Lyal 1999, Lü and Zhang 2007). Some of these weevils are significant pests in agroforestry systems, as their larvae feed on stems, fruits, seeds, or other tissues of host plants (Lyal 2014, Abdulla et al. 2016, Zhang 2016, Jeger et al. 2018, Hsiao and Oberprieler 2020). Therefore, the rapid and accurate identification of species will expedite efforts in ecology, taxonomy, plant protection, and quarantine.

The selection of molecular delimitation methods is critical in species delimitation and biodiversity assessment (Ge et al. 2021), as different algorithmic principles employed by various methods may not yield consistent delimitations of putative species (Song et al. 2018). The Barcode Index Number (BIN) system utilizes various distance metrics to reconstruct a neighbor-joining tree, subsequently establishing a persistent registry for organisms in the Barcode of Life Data System (BOLD) (www.bold.system.org) (Ratnasingham and Hebert 2013). The Automatic Barcode Gap Discovery (ABGD) algorithm identifies a divergence gap that corresponds to the differentiation between intraspecific and interspecific distances (Puillandre et al. 2012). Assemble Species by Automatic Partitioning (ASAP), proposed by Puillandre et al. in 2021, is a new method to build species partitions from single locus sequence alignments. The Poisson tree processes (PTP) can give a species delimitation hypothesis based on gene trees inferred from molecular sequences (Zhang et al. 2013). The Bayesian Poisson tree processes (bPTP) is a Bayesian implementation of the PTP model (Zhang et al. 2013). Additionally, jMOTU can efficiently and robustly identify the insect groups present in survey datasets within a short timeframe based on molecular classification (Jones et al. 2011).

In this study, we have developed a DNA barcode dataset comprising 255 morphospecies and 1,290 sequences. Subsequently, we conducted a comprehensive analysis that included genetic distance (GD), species delimitation, and population structure to investigate the cryptic species diversity of the weevils of Molytinae.

## Materials and Methods

### Sample Collection and Identification

The specimens of Molytinae weevils were collected in various Chinese provinces, including Beijing, Hunan, Hubei, Yunnan, Tibet, and Inner Mongolia from 2018 to 2021 (Supplementary Table S1). Latitude and longitude coordinates were recorded for each collection site. All specimens were preserved in 95% ethanol and stored at −20 °C until further use. All collected specimens were morphologically identified by Li Ren and Jinliang Ren with literature (Chao and Chen 1980, Alonso-Zarazaga et al. 2023). All voucher specimens were deposited in the Institute of Zoology (IZCAS), Chinese Academy of Sciences, Beijing, China.

### DNA Extraction, Amplification, and Sequencing

We utilized DNeasy Blood & Tissue Kits (QIAGEN, Germany) to extract DNA from all specimens. Depending on the specimen size, DNA extraction was performed from 3 legs on the right side or the entire body (Ma et al. 2022). The amplifications of COI sequences through polymerase chain reaction (PCR) were performed using the primers LCO1490 (GGTCAACAAATCATAAAGATATTGG) and HCO2198 (TAAACTTCAGGGTGACCAAAAAATCA) (Folmer et al. 1994). PCR mixes (25 ml) contained 12.5 μl 2× Taq PCR MasterMix (Tiangen Biotech Co., Ltd., Beijing, China), 1 μl of forward and reverse primers each (Sangon Biotech Co. Ltd., Shanghai, China), 2 μl of total undiluted DNA template, and 8.5 μl of ddH_2_O. PCR profile is as follows: 94 °C for 2 min, first cycle set (5 repeats): 94 °C for 40 s, 45 °C for 40 s, and 72 °C for 60 s. Second cycle set (35 repeats): 94 °C for 40 s, 51 °C for 40 s, and 72 °C for 60 s, followed by elongation at 75 °C for 5 min (Ma et al. 2022). PCR products were visualized by performing 1% agarose gel electrophoresis in TAE (Tris-acetate-EDTA) buffer. Subsequently, the successful PCR products were submitted to the Beijing Genomics Institute (Shenzhen, China) for sequencing. The raw data were compiled and edited using SeqMan software (ver. 7.1). Barcode sequences, along with voucher specimen information, were uploaded to the BOLD database.

### Publicly Available Data

We retrieved sequences of species of Molytinae (up to August 2023) from the BOLD database. To refine our dataset, we applied filters to exclude sequences that were (i) shorter than 600 bp, (ii) containing degenerate and missing bases, (iii) having degenerate bases, and (iv) unidentified at the species level. The removal of duplicate sequences was performed for each species. Subsequently, all sequences were subjected to translation into amino acids using MEGA ver. 7 in order to verify and prevent the occurrence of stop codons.

### Data Integration and GD Analysis

The dataset utilized in this study comprises sequences obtained from BOLD and those generated internally. To ensure accuracy, multiple sequence alignment and pruning were conducted using the online version of MAFFT ver. 7 with default parameters (Katoh and Standley 2013). We employed the MEGA ver. 7 software and utilized the Kimura 2-parameter (K2P) model to calculate intraspecific GDs (intra-GD) and interspecific GDs (inter-GD) (Kimura 1980, Kumar et al. 2016). Subsequently, we utilized Origin 2018 software to visually represent the distribution of GDs through histograms and scatter plots (Moberly et al. 2018).

### Phylogenetic Analysis and Molecular Species Delimitation

The phylogenetic inference analyses were conducted using maximum likelihood (ML). The optimal nucleotide substitution models and ML analyses were selected based on the Akaike information criterion through the IQ-TREE web server (Trifinopoulos et al. 2016). The ML analyses were performed with the following settings: ML + Ultrafast bootstrap, 1,000 replicates, and the GTR + F + I + G4 model. The obtained trees were visualized and edited using iTOL (Letunic and Bork 2016). The dataset sequences were condensed into unique haplotypes using DNAsp ver. 5 (Librado and Rozas 2009). Haplotype network analysis for some species complexes was constructed using TCS in POPART (Clement et al. 2009, Leigh and Bryant 2015).

The 6 species delimitation methods, namely ABGD, ASAP, BIN, PTP, bPTP, and jMOTU, were employed to identify potential molecularly operational taxonomic units (MOTUs). The ABGD analyses were conducted using the web server (https://bioinfo.mnhn.fr/abi/public/abgd/abgdweb.html) (Puillandre et al. 2012), with a relative gap width of *X* = 1.0, K2P distance metric, intraspecific divergence (P) values ranging from 0.005 to 0.1, while keeping other parameters at their default settings. The ASAP analyses were performed at the web server (https://bioinfo.mnhn.fr/abi/public/asap/asapweb.html) (Puillandre et al. 2021), K2P distance metric, choose the result with the lowest score. The BIN number was directly queried and tallied during the analysis (Ratnasingham and Hebert 2013). The PTP analyses were performed on the web server (https://mptp.h-its.org/#/tree), with all parameters at their default settings. The bPTP analyses were performed on the web server (http://species.h-its.org/ptp/) using the following setting: 500,000 **Markov Chain Monte–Carlo **(MCMC) generations, with the initial 20% of trees discarded as burn-in (Zhang et al. 2013). The jMOTU analyses were performed at the analytical package with parameters to use for MOTU definition 1 to 50, a low BLAST identity filter at 97%, percentage of minimum sequence length at 95%, and number of processors to use in Magablast at 4 (Jones et al. 2011).

When 3 or more species delimitation methods grouped sequences of a morphospecies into a single MOTU, we considered the results of both morphological identification and molecular species delimitation to be congruent; when sequences of the same morphospecies were assigned to multiple MOTUs by 4 or more species delimitation methods, or different morphospecies were grouped into a single MOTU, we regarded these instances as “taxonomic warnings” (Li et al. 2023).

## Results

The complete dataset comprised 1,290 barcodes from 255 species within 89 genera (Supplementary Table S2). The analysis revealed that out of the examined species, 112 (43.9%) had a single sequence, while 37 (14.5%) exhibited only 2 sequences, conversely, more than 2 sequences were obtained for 106 (41.6%) species. Notably, *Conotrachelus* comprised the largest number with 28 species and 179 sequences. The highest number of sequences per species was accounted for by *Typoderus admetus* with a total of 55 sequences. The dataset yielded a total of 742 unique haplotypes (Supplementary Table S3).

### Genetic Distance

**Fig. 1.**
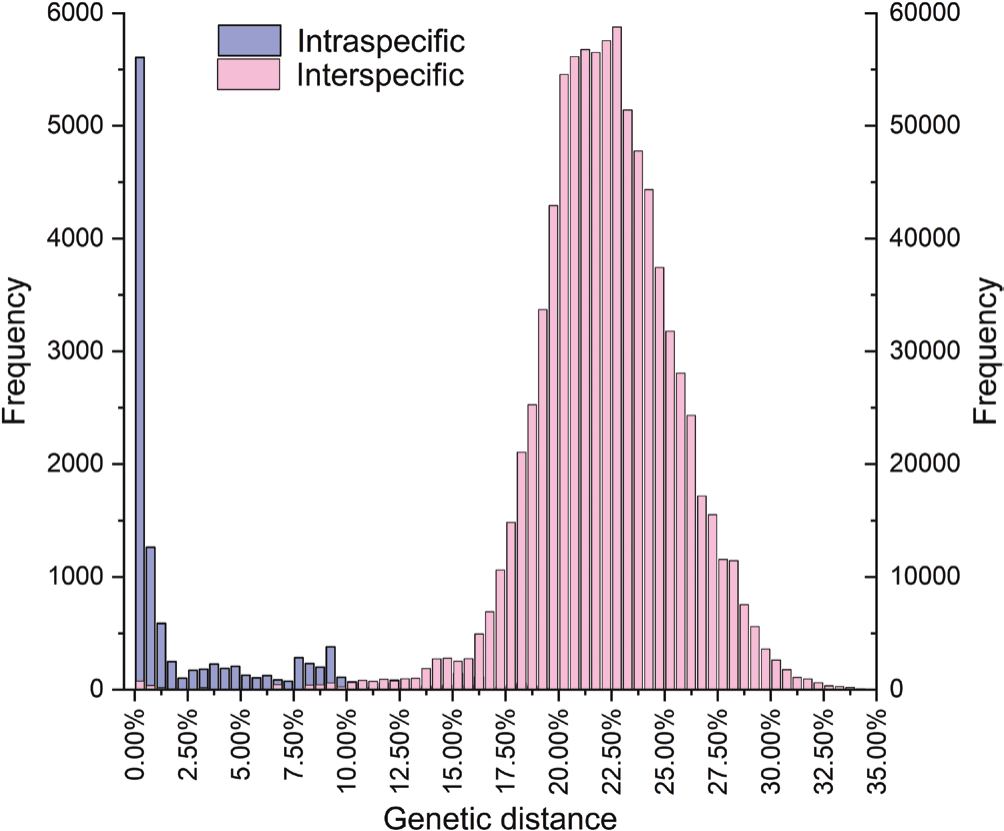
Intra- and inter-GD histograms of species of Molytinae.

Our results showed that the intra-GD ranged from 0% to 27.50% with an average of 2.90%, while the inter-GD varied between 0% and 39.30% with an average of 22.26% (Fig. 1). The maximum intraspecific divergence observed in *Aclees cribratus* reached 27.50%, surpassing the average inter-GD within our dataset. Surprisingly, several species groups, *Hylobius pinicola* and *H. warreni* (0.56%–1.13%), *Ornatalcides trifidu* and *Sternuchopsis trifida* (0%–1.3%), *Aeatus costulatus* and *A.* sp. cur263SG and *A.* sp. cur242SG (1.31%–3.95%), *Lepyrus nordenskioeldi* and *L. stefanssoni* (0.19%–11.94%), and *Melanterius* sp. SPN59 and *M.* sp. SPN60 (1.50%–1.70%) exhibited lower inter-GD values. The maximum inter-GD (39.30%) was observed between *Tazarcus aeaea* and *Araeoscapus* sp. NZAC 03037707. Notably, the minimum inter-GD of 123 species is higher than the maximum intra-GD observed within this study. Specifically, the intra-GD of 97 species was found to be below the threshold value of 3.00%, while the inter-GD of 220 species exceeded this threshold.

### Species Delimitation

**Fig. 2.**
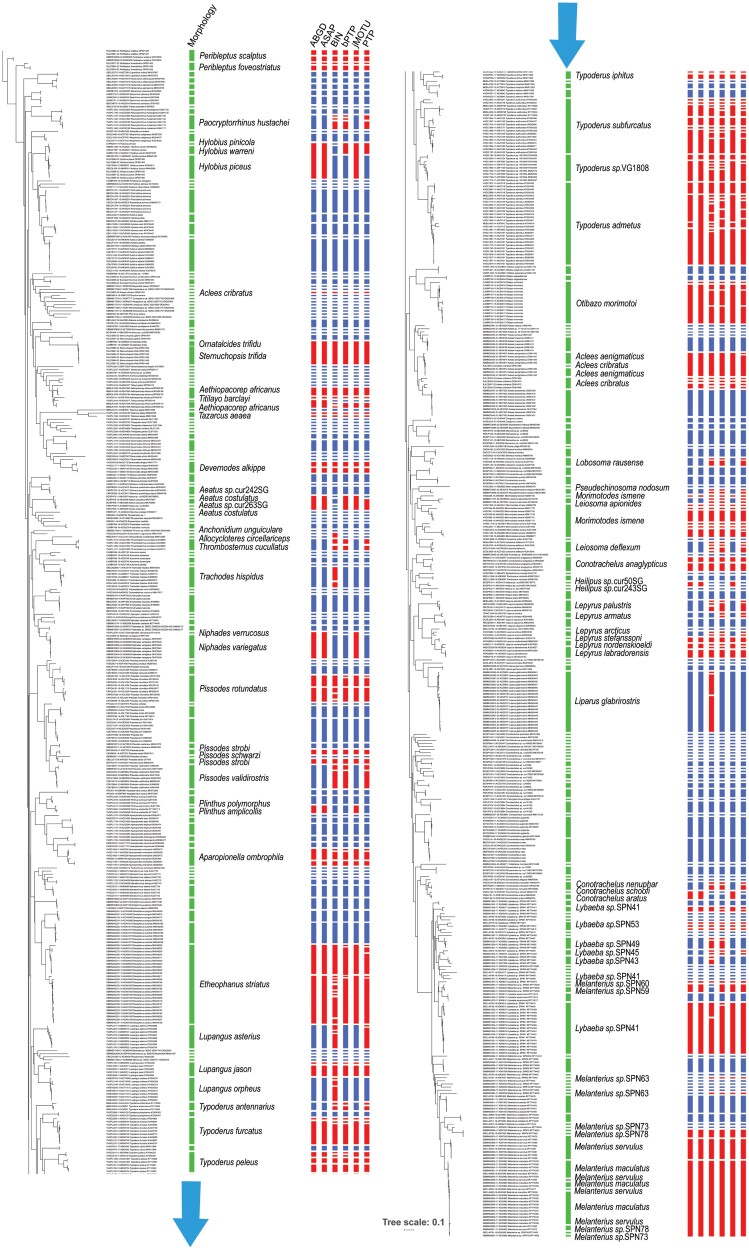
Delimitation of 255 species of Molytinae based on morphology, ABGD, ASAP, BIN, bPTP, jMOTU, and PTP are shown by bars next to species names.

The number of MOTUs varied from 275 to 494, depending on the employed methodologies (Fig. 2). The ASAP and ABGD analyses yielded a relatively conservative result of 275 and 279 MOTUs, respectively, among which taxonomic concordance was observed for 211 MOTUs when compared with the obtained results of morphological identification. The jMOTU method successfully identified a total of 279 MOTUs, which closely matched the numbers of predetermined morphospecies. Among these, 206 MOTUs corresponded with the morphospecies that were previously identified. The PTP and bPTP analyses identified a total of 322 and 320 MOTUs, respectively, with approximately 201 and 200 MOTUs showing consistent classification compared with the morphological classification. The BIN method yielded the largest number of MOTU, 200 MOTUs consistent with morphological results. Out of the total 255 morphospecies identified based on their morphological characteristics, a significant proportion of 83.1% (212) species were unambiguously delimited by their COI sequences using various species delimitation methods (Fig. 2).

### Investigation on Taxonomic Warnings

**Fig. 3.**
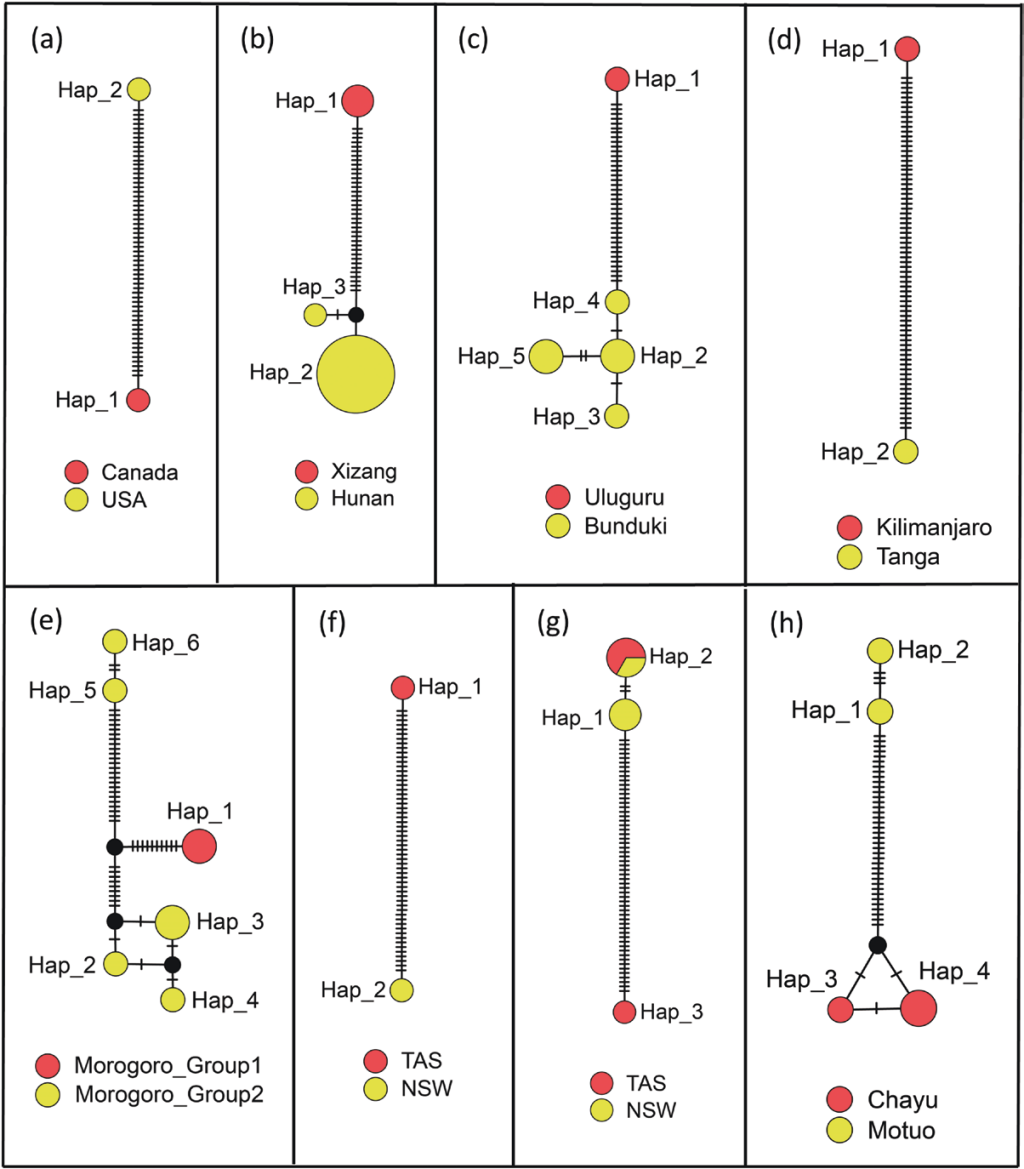
Haplotype networks for species with multiple MOTUs based on COI sequences. A) *Lepyrus nordenskioeldi*; B) *Peribleptus foveostriatus*; C) *Lupangus jason*; D) *Tazarcus aeaea*; E) *Aparopionella ombrophila*; F) *Melanterius* sp. SPN73; G) *Melanterius* sp. SPN78; H) *Peribleptus scalptus*. These species showed different geographical distributions. The circles represent different haplotypes, and the short line segments indicate mutated positions between haplotypes. Different sizes of the circles represent geographical regions and relative numbers of sequences.

Our research has uncovered certain inconsistencies between molecular and morphospecies, specifically in terms of different methods used for species delineation. As a result, a total of 28 morphological species were found to be assigned to more than one MOTU (Table 1). A total of 21 species exhibited population divergence based on geographical distribution, including *Lepyrus nordenskioeldi*, *Peribleptus foveostriatus*, *Lupangus jason*, *Lachnus roboris*, *Tazarcus aeaea*, *Aparopionella ombrophila*, *Melanterius* sp. SPN73, and *P. scalptus* (Fig. 3; Supplementary Fig. S1). The remaining 7 species, namely *Typoderus admetus*, *Lybaeba* sp. SPN41, *Devernodes alkippe*, *Etheophanus striatus*, *Melanterius* sp. SPN63, *T. iphitus*, and *Conotrachelus anaglypticus*, exhibited no discernible genetic patterns based on geographical distribution (Supplementary Fig. S1). Furthermore, 3 groups of species demonstrated the most consistent inter-GD values, resulting in their classification as a single MOTU across all 4 species delimitation methods. The inter-GDs were found to be low within 2 species (*Ornatalcides trifidu* and *Sternuchopsis trifida*, 0%–1.3%) were identified.

**Table 1.** Thirty-eight morphospecies containing possible cryptic diversity or population divergence indicated by more than 4 of the 6 species delimitation methods

Morphospecies	Sequence number	MOTU						The max-intra-GD
		ABGD	ASAP	BIN	bPTP	jMOTU	PTP	
*Aclees cribratus*	7	5	5	6	6	6	6	27.5%
*Aethiopacorep africanus*	6	3	3	4	3	3	3	10.7%
*Aparopionella ombrophila*	8	2	2	3	3	2	3	7.1%
*Conotrachelus anaglypticus*	11	2	2	2	2	2	2	12.3%
*Devernodes alkippe*	12	2	2	2	2	2	2	8.6%
*Etheophanus striatus*	50	2	2	9	4	2	5	11.2%
*Lepyrus labradorensis*	16	2	2	2	2	2	2	7.6%
*Lepyrus nordenskioeldi*	2	2	2	2	2	2	2	12.2%
*Lupangus jason*	7	2	2	2	2	2	2	7.9%
*Lybaeba* sp. SPN41	26	3	3	4	5	3	4	14.2%
*Lybaeba* sp. SPN53	3	3	3	3	3	3	3	12.6%
*Melanterius servulus*	28	3	3	3	3	3	3	15.3%
*Melanterius* sp. SPN63	2	2	2	2	2	2	2	17.6%
*Melanterius* sp. SPN73	2	2	2	2	2	2	2	11.5%
*Melanterius* sp. SPN78	6	2	2	2	2	2	2	11.7%
*Morimotodes ismene*	34	7	7	7	7	7	7	19.1%
*Otibazo morimotoi*	17	2	2	4	4	2	4	15.5%
*Peribleptus foveostriatus*	15	2	2	2	2	2	2	7.5%
*Peribleptus scalptus*	7	2	2	2	2	2	2	8.3%
*Pissodes rotundatus*	9	2	2	4	3	2	3	13.2%
*Pissodes strobi*	4	2	2	2	2	2	2	8.2%
*Tazarcus aeaea*	2	2	2	2	2	2	2	15.1%
*Typoderus admetus*	55	4	2	10	6	3	6	10.7%
*Typoderus furcatus*	13	2	2	3	3	2	3	8.0%
*Typoderus iphitus*	3	2	2	2	2	2	2	10.6%
*Typoderus peleus*	12	3	3	4	4	3	3	12.7%
*Typoderus* sp. VG1808	12	2	2	5	5	2	3	12.1%
*Typoderus subfurcatus*	24	6	6	9	10	6	10	18.3%

## Discussion

### Molecular Species Delimitation of Molytinae

The utilization of precise molecular species delimitation methods can expedite the process of biodiversity assessment, which holds particular significance within the realm of highly diverse insect taxa (Song et al. 2018, Rodrigues and Galati 2023). The ASAP method produced the most conservative results compared with jMOTU, PTP, bPTP, BIN, and ABGD when applied to a dataset of 255 Molytinae morphological species. Consistent with previous research, jMOTU, ASAP, and ABGD yielded a relatively conservative number of MOTUs (Song et al. 2018, Ge et al. 2021, Li et al. 2023, Hsiao and Oberprieler 2024). The difference in algorithmic principles may account for this phenomenon. The jMOTU method was specifically designed to optimize global comparison using the Needleman–Wunsch algorithm and generate OTUs based on user-selected cutoff values (Jones et al. 2011, Song et al. 2018). The analytical package jMOTU yielded 279 MOTUs when employing single clustering and a selected cutoff of 29 (5.41%). The ABGD method demonstrates superior performance when the level of intraspecific variation is lower than interspecific variation (Puillandre et al. 2012). The ABGD method, consistent with the findings of previous research (Hsiao and Oberprieler 2024), did not yield the most conservative outcomes in this study. After re-evaluating our data again, we have determined that this discrepancy is attributed to the fact that the maximum intra-GD exceeds the minimum inter-GD in certain species. The BIN method yielded unsatisfactory results due to the presence of a high percentage of 55 species with an intra-GD exceeding 2.2%. Conversely, the bPTP method demonstrates greater sensitivity toward imbalanced sample sizes within the dataset (Zhang et al. 2013). The dataset of Molytinae exhibited a sampling bias, with only 43.9% of species represented by a single sequence, while 14.1% had 10 or more sequences, and even *Typoderus admetus* was represented by as many as 55 sequences. The presence of this sampling bias may account for the suboptimal performance observed in the bPTP method.

A typical characteristic observed in datasets containing barcodes is that the minimum inter-GD exceeds the maximum intra-GD (Ma et al. 2022). In this context, a well-defined “threshold” can facilitate the discovery of cryptic species or new species (Song et al. 2018, Ma et al. 2022). There is no universally applied threshold for all taxa due to variations in population sizes and the timing of species differentiation (Greenstone et al. 2011, Pramual and Adler 2014). For example, different thresholds have been suggested for certain groups within Hemiptera (5.00%) (Park et al. 2011), Coleoptera (9.18%) (Ma et al. 2022), and Diptera (4%–5%) (Lin et al. 2015). In our study, 73.0% of morphological species can be accurately defined when the GD threshold is set at 3%. It is evident that the utilization of a GD threshold solely as an empirical method necessitates further incorporation of scientific algorithms for species recognition (Li et al. 2023). The species boundaries of Molytinae were effectively delineated using 4 methods (ABGD, BIN, bPTP, and jMOTU), resulting in unambiguous discrimination of 89.0% (227) species. This outcome significantly outperformed the fixed threshold approach.

### Taxonomic Warnings in Molytinae

The haplotype network analyses conducted in this study revealed the existence of multiple MOTUs within certain species, which can be attributed to their distinct geographical distributions and potentially indicate the presence of cryptic species (Fig. 3, Supplementary Fig. S1). The *Lepyrus nordenskioeldi* species can be classified into 2 MOTUs according to 4 molecular delimitation methods (Fig. 3A). These MOTUs, one found in Alaska, USA, and the other in Nunavut, Canada, exhibited significant genetic divergence of 12.2%. The long-term geographic isolation of *L. nordenskioeldi* may result in the differentiation of distinct genetic lineages, potentially indicating the presence of a cryptic species within this taxon. The remaining 20 species, including *Peribleptus foveostriatus*, exhibited similar patterns. Specifically, *P. foveostriatus* represented by 2 MOTUs in all molecular delimitation methods: one from Xizang of China and another from Hunan (Fig. 3B). These 2 MOTUs displayed a larger genetic divergence of 7.5%.

The theory of shifting balance suggests that numerous species consist of small, partially isolated populations (Slatkin 1987, Song et al. 2018). Therefore, geographic populations exhibiting profound intraspecies variations are more likely to unveil cryptic species, thereby suggesting the presence of potential cryptic species within the weevils of Molytinae. The distribution patterns of enigmatic genetic lineages, such as *Typoderus admetus*, *Lybaeba* sp. SPN41, *Devernodes alkippe*, *Etheophanus striatus*, *Melanterius* sp. SPN63, *Typoderus iphitus*, and *Conotrachelus anaglypticus*, did not exhibit discernible geographical trends in our findings. The *Devernodes alkippe* found in the Emei Shan region of China, which is located in the northern section of the Hengduan mountains (Li et al. 2020), serves as a prime example. The abundance of cryptic diversity of *D. alkippe* may be primarily driven by significant environmental heterogeneity and the presence of ecologically distinct habitats (Xing and Ree 2017, Li et al. 2019).

We observed that different species delimitation methods consistently assigned some groups of species of Molytinae to the same MOTU, and some of these groups exhibited smaller minimum inter-GD, such as *Ornatalcides trifidu* and *Sternuchopsis trifida*. These 2 species exhibited a low inter-GD (0%–1.3%) and were assigned a single MOTU. Pascoe (1870) initially described *Alcides trifidus*. Pajni and Dhir (1987) proposed the establishment of the genus *Mesalcidodes* Voss 1958, designating *M. trifidus* (Pascoe) as the type species subsequently. However, Alonso-Zarazaga and Lyal (1999) referred to this species as *Ornatalcides trifidus*. In the latest works, Alonso-Zarazaga et al. (2023) proposed that *Mesalcidodes* are considered a subgenus of *Sternuchopsis* and that *O. trifidus* are referred to as *S. trifida*. The occurrence of *S. trifida* has been documented in both China and Japan (Frye et al. 2014). Based on the comparison of specimen images of *O. trifidu* from BOLD database and voucher specimen of *S. trifida*, we concluded that both belong to the same species, namely *S. trifida*. Similarly, with their low interspecific GD, other species groups, *Hylobius pinicola* and *H. warreni*, *Aeatus costulatus*, *A.* sp. cur263SG and *A.* sp. cur242SG, *Lepyrus nordenskioeldi* and *L. stefanssoni*, and *Melanterius* sp. SPN59 and *M.* sp. SPN60, and *M. maculatus*, *M. servulus*, *M.* sp. SPN73 and *M.* sp. SPN78 were all or part grouped into one MOTU by 4 or more molecular methods. Taking *Hylobius pinicola* and *H. warreni* as an example, there are 5 molecular methods to assign them as an MOTU. Based on the available evidence, we believe an identification error has occurred. Without access to the relevant specimens, we cannot determine which party was misidentified.

## Conclusion and Prospects

In general, DNA barcodes can successfully delimit species of Molytinae with 86.6% match with morphospecies. Comparing the performances of different analytical tools, the ASAP method fits well with the COI barcode dataset of Molytinae. Unusual deep intraspecific divergences in some species complexes were detected, indicating potential morphological misidentifications, population divergence, or cryptic species. It is urgent to incorporate additional nuclear genes, more morphological traits (e.g., male genitalia), and ecological data for further research. The combination of morphology and DNA-based taxonomy is crucial for biodiversity surveys.

## Supplementary Material

ieae083_suppl_Supplementary_Tables

ieae083_suppl_Supplementary_Figure_S1
