# Supplementary material for: Delimiting species, revealing cryptic diversity in Molytinae (Coleoptera: Curculionidae) weevil through DNA barcoding
Source: J Insect Sci. 2024 Sep 30;24(4):25. doi: 10.1093/jisesa/ieae083 (PMC11441576; doi:10.1093/jisesa/ieae083)
Supplement: ieae083_suppl_Supplementary_Figure_S1 [file ieae083_suppl_supplementary_figure_s1.pdf]

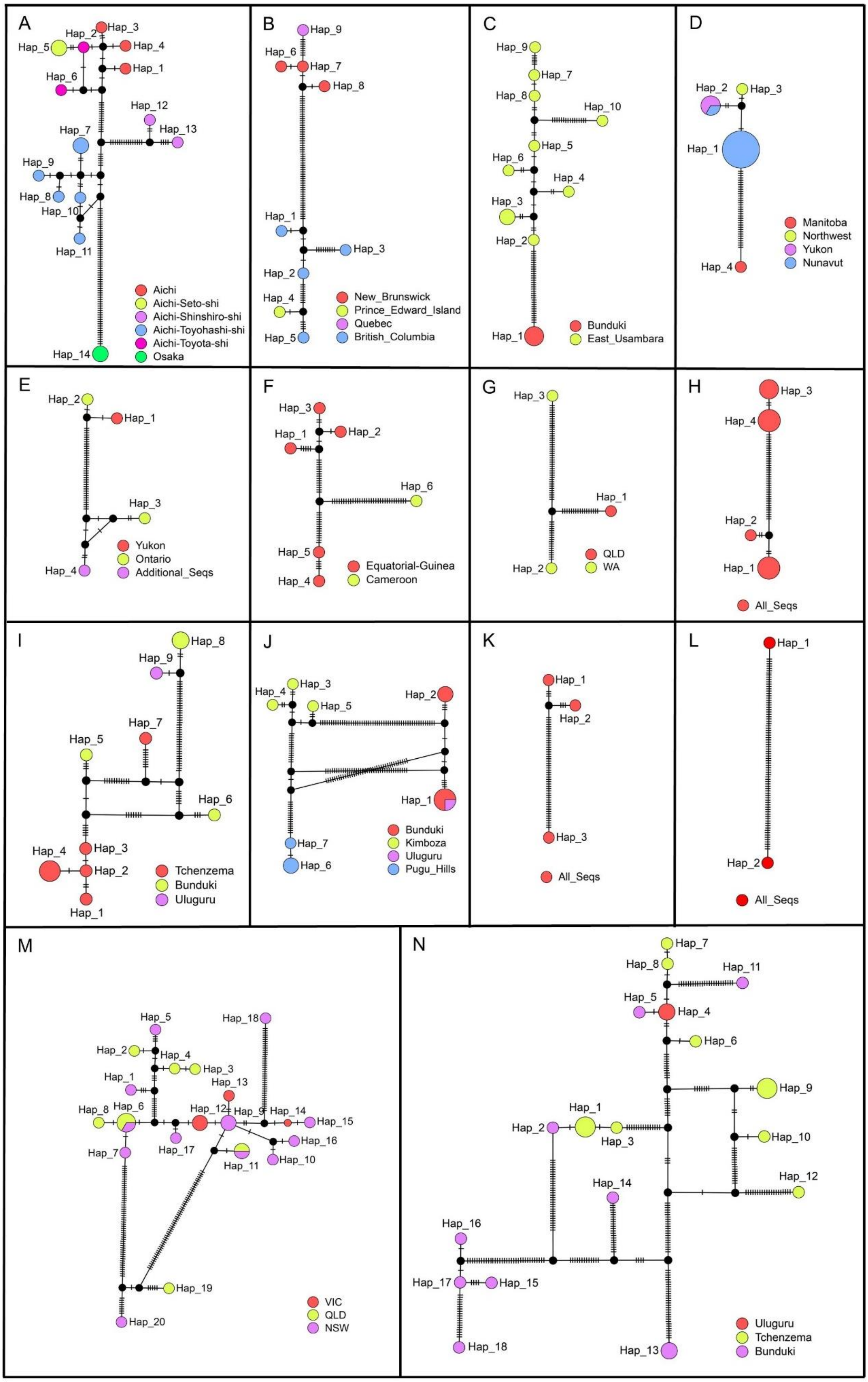
A
Hap_3
Hap_2
Hap_5
Hap_4
Hap_1
Hap_6
Hap_12
Hap_7
Hap_13
Hap_9
Hap_8
Hap_10
Hap_11
Hap_14
Aichi
Aichi-Seto-shi
Aichi-Shinshiro-shi
Aichi-Toyohashi-shi
Aichi-Toyota-shi
Osaka
B
Hap_9
Hap_6
Hap_7
Hap_8
Hap_1
Hap_3
Hap_2
Hap_4
Hap_5
New_Brunswick
Prince_Edward_Island
Quebec
British_Columbia
C
Hap_9
Hap_7
Hap_8
Hap_10
Hap_5
Hap_6
Hap_4
Hap_3
Hap_2
Hap_1
Bunduki
East_Usambara
D
Hap_2
Hap_3
Hap_1
Hap_4
Manitoba
Northwest
Yukon
Nunavut
E
Hap_2
Hap_1
Hap_3
Hap_4
Yukon
Ontario
Additional_Seqs
F
Hap_3
Hap_1
Hap_2
Hap_6
Hap_5
Hap_4
Equatorial-Guinea
Cameroon
G
Hap_3
Hap_1
Hap_2
QLD
WA
H
Hap_3
Hap_4
Hap_2
Hap_1
All_Seqs
I
Hap_8
Hap_9
Hap_7
Hap_5
Hap_6
Hap_4
Hap_3
Hap_2
Hap_1
Tchenzema
Bunduki
Uluguru
J
Hap_3
Hap_4
Hap_5
Hap_2
Hap_1
Hap_7
Hap_6
Bunduki
Kimboza
Uluguru
Pugu_Hills
K
Hap_1
Hap_2
Hap_3
All_Seqs
L
Hap_1
Hap_2
All_Seqs
M
Hap_5
Hap_18
Hap_2
Hap_4
Hap_3
Hap_1
Hap_13
Hap_8
Hap_6
Hap_12
Hap_9
Hap_14
Hap_15
Hap_16
Hap_7
Hap_17
Hap_11
Hap_10
Hap_19
Hap_20
VIC
QLD
NSW
N
Hap_7
Hap_8
Hap_11
Hap_5
Hap_4
Hap_6
Hap_9
Hap_1
Hap_2
Hap_3
Hap_10
Hap_14
Hap_12
Hap_16
Hap_17
Hap_15
Hap_18
Hap_13
Uluguru
Tchenzema
Bunduki

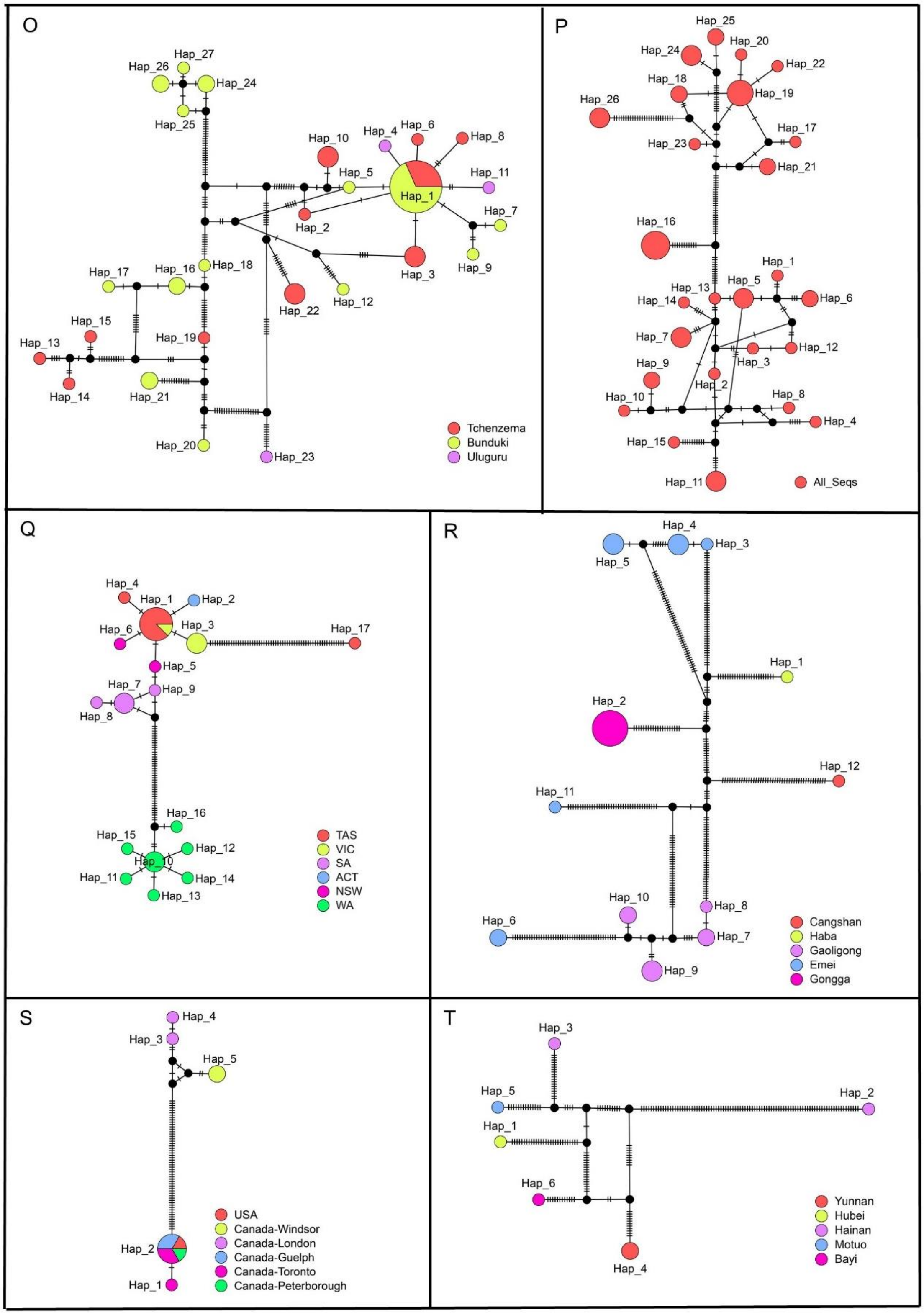

Figure S1. Haplotype networks for species with multiple MOTUs based on COI sequences. (A) *Otibazo morimotoi*; (B) *Pissodes rotundatus*; (C) *Typoderus furcatus*; (D) *Lepyrus labradorensis*; (E) *Pissodes strobi*; (F) *Aethiopacorep africanus*; (G) *Lybaeba* sp. SPN53; (H) *Devernodes alkippe*; (I) *Typoderus* sp. VG1808; (J) *Typoderus peleus*; (K) *Typoderus iphitus*; (L) *Melanterius* sp. SPN63; (M) *Lybaeba* sp. SPN41; (N) *Typoderus subfurcatus*; (O) *Typoderus admetus*; (P) *Etheophanus striatus*; (Q) *Melanterius servulus*; (R) *Morimotodes ismene*; (S) *Conotrachelus anaglypticus*; (T) *Aclees cribratus*. The circles represent different haplotypes, and the short line segments indicate mutated positions between haplotypes. Different

colours and sizes of the circles represent geographical regions and relative numbers of sequences.
